# The integration of epigenomic and transcriptomic sequencing data reveals cell‐specific Bexarotene‐induced transcriptional activity in APP/PS1 mouse brain

**DOI:** 10.1002/alz.095714

**Published:** 2025-01-09

**Authors:** Yi Lu, Carolina Saibro‐Girardi, Nicholas F Fitz, Iliya Lefterov, Radosveta Koldamova

**Affiliations:** ^1^ University of Pittsburgh, Pittsburgh, PA USA

## Abstract

**Background:**

Treatment with the RXR‐specific agonist Bexarotene exerts neuroprotective effects in Alzheimer’s disease (AD) mouse models by improving cognition and increasing Aβ clearance. At the transcriptional level, ligand‐activated RXR receptors regulate gene networks linked to neural development, neuroinflammation, and metabolism. This study aimed to reveal the association between changes in chromatin architecture and transcriptional activity in the brain of Bexarotene‐treated APP/PS1 mice.

**Methods:**

APP/PS1D9 mice were treated orally with bexarotene or vehicle (corn oil, DMSO 1%). Mouse brains were dissociated and used for cDNA library generation on a 10x Genomics platform for single‐cell resolution. *Seurat* pipeline was performed to resolve cell populations and for differential expression analysis, followed by GO analysis to reveal cell population‐specific transcriptional response to Bexarotene. Changes in chromatin architecture were evaluated by single‐cell ATAC‐seq. scATAC‐seq data were processed and integrated with scRNA‐seq using the *ArchR* pipeline.

**Results:**

We present a single‐cell‐resolution map of transcriptomic and epigenomic changes in Bexarotene‐treated mice. We profiled transcriptional and epigenomic landscapes of 37,640 cells (scRNA‐seq) and 61,353 individual nuclei (snATAC‐seq) from 8 samples (4 Bexa and 4 vehicle controls). To predict candidate upstream regulators that may be shaping the chromatin accessibility landscape of brain cells following ligand activation of RXR and contributing to cell type identity and cell fate specification, we used chromVar. We discovered 67 high‐confidence candidate regulators that show strong correlations between TF enrichment and ATAC‐inferred expression and between ATAC‐inferred expression and RNA‐measured expression. Next, we used the integrated single cell RNA‐seq and ATAC‐seq data to identify cell type‐ and disease‐specific *cis*‐regulatory elements (CREs) and their target genes by correlating gene expression with chromatin accessibility across all nuclei in the dataset. We then asked if the differential gene expression we observed could be mediated by candidate CREs. The majority of the DEGs identified between Bexa and control nuclei had a linked peak in the same cell type where the gene was differentially expressed. An example is *Apoe* gene located in the *Apoe/Apoc* locus that was significantly upregulated by Bexa in microglia but not in astrocytes.

**Conclusions:**

Results revealed that Bexarotene affected chromatin accessibility in microglia in correlation with *Apoe* expression.

